# Alpine Grassland Soil Organic Carbon Stock and Its Uncertainty in the Three Rivers Source Region of the Tibetan Plateau

**DOI:** 10.1371/journal.pone.0097140

**Published:** 2014-05-12

**Authors:** Xiaofeng Chang, Shiping Wang, Shujuan Cui, Xiaoxue Zhu, Caiyun Luo, Zhenhua Zhang, Andreas Wilkes

**Affiliations:** 1 State Key Laboratory of Soil Erosion and Dryland Farming on Loess Plateau, Institute of Soil and Water Conservation, Northwest A&F University, Yangling, China; 2 Key Laboratory of Adaptation and Evolution of Plateau Biota, Northwest Institute of Plateau Biology, Chinese Academy of Science, Xining, China; 3 Laboratory of Alpine Ecology and Biodiversity, Institute of Tibetan Plateau Research, Chinese Academy of Sciences, Beijing, China; 4 University of Chinese Academy of Science, Beijing, China; 5 World Agroforestry Centre East Asia Programme, Beijing, China; USDA-ARS United States of America

## Abstract

Alpine grassland of the Tibetan Plateau is an important component of global soil organic carbon (SOC) stocks, but insufficient field observations and large spatial heterogeneity leads to great uncertainty in their estimation. In the Three Rivers Source Region (TRSR), alpine grasslands account for more than 75% of the total area. However, the regional carbon (C) stock estimate and their uncertainty have seldom been tested. Here we quantified the regional SOC stock and its uncertainty using 298 soil profiles surveyed from 35 sites across the TRSR during 2006–2008. We showed that the upper soil (0–30 cm depth) in alpine grasslands of the TRSR stores 2.03 Pg C, with a 95% confidence interval ranging from 1.25 to 2.81 Pg C. Alpine meadow soils comprised 73% (i.e. 1.48 Pg C) of the regional SOC estimate, but had the greatest uncertainty at 51%. The statistical power to detect a deviation of 10% uncertainty in grassland C stock was less than 0.50. The required sample size to detect this deviation at a power of 90% was about 6–7 times more than the number of sample sites surveyed. Comparison of our observed SOC density with the corresponding values from the dataset of Yang et al. indicates that these two datasets are comparable. The combined dataset did not reduce the uncertainty in the estimate of the regional grassland soil C stock. This result could be mainly explained by the underrepresentation of sampling sites in large areas with poor accessibility. Further research to improve the regional SOC stock estimate should optimize sampling strategy by considering the number of samples and their spatial distribution.

## Introduction

Soil stores more carbon (C) than the vegetation and atmosphere pools combined, and minor changes in soil organic carbon (SOC) stock could have momentous effects on atmospheric CO_2_ concentrations [1]. As the Earth's third pole, the Tibetan Plateau is mostly covered by typical alpine grasslands, which contain large soil C stocks [2], [3]. Alpine grasslands in the Tibetan Plateau could feedback to accelerate the current warming trend by releasing large amounts of this stored C to the atmosphere [4], [5]. Therefore, estimates of organic C stocks in alpine grasslands are crucial for understanding the regional and global greenhouse gas balance [2]. Despite considerable research over the past 20 years, much uncertainty exists regarding the SOC stock in the alpine grasslands. For example, Yang et al. [2] used a satellite-based approach and estimated that the SOC stock in the top 1 m in alpine grasslands was 7.4 Pg C, with an average density of 6.5 kg C m^−2^. Wang et al. [3], using the First National Soil Survey dataset and field measurements surveyed in the eastern part of the Tibetan Plateau, estimated the SOC stock at 33.52 Pg for alpine grasslands, with an average SOC density of 20.9 kg C m^−2^. Therefore, precise quantification of soil C stocks in alpine grasslands of the region is needed to make credible conclusions about the potential scale of feedback between the terrestrial C cycle and climate.

Regional scale assessments of SOC have typically been supported by data from soil inventories [3]. An important issue with soil C stock inventories is spatial heterogeneity [6]. Increased SOC variability causes decreased sampling representativeness and increased sample size is needed to estimate the true SOC distribution [7], [8]. Previous studies also found that a large number of sampling plots is useful to assess the spatial variation of C stocks in a heterogeneous landscape and to reduce the uncertainty in the final SOC estimates [9]. Yu et al. [10] examined spatial variability of SOC in a red soil region of South China varying in land use and soil type, using six sampling densities (14, 34, 68, 130, 255 and 525 points in 927 km^2^). They found that high sampling densities gradually decreased the variation in SOC. Similarly, Muukkonen et al. [11] showed that the spatial variation in C stock in boreal forest soil decreased with increasing number of samples, without further increase in the precision of the estimate after 20–30 samples in a 6.25 m^2^ area. Such results suggest incentives for soil studies to increase the number of samples to reduce variability and improve soil C stock estimates. Despite these small–scale efforts, there is a lack of information on the effects of sampling effort on SOC estimates at regional scale [10], [12].

As the variability within a relatively homogeneous stratum is lower than the variability within a broad heterogeneous landscape, stratification of soil sampling can improve the SOC stock estimate [13], [14]. These results were the basis for conducting a stratified, random sampling design in the present study. Because grassland type is the most important variable driving the spatial pattern of SOC [15], the Three Rivers Source Region (TRSR) was stratified by grassland type, and this strategy is expected to capture a large part of SOC variation. In this study, we assessed alpine grassland soil C stock and its uncertainty. Specifically, our study objectives were (i) to quantify SOC density from alpine grasslands at three depths (0–10, 0–20 and 0–30 cm), and (ii) to investigate the statistical power and sample size requirement to detect a deviation of 10% uncertainty.

## Materials and Methods

### Ethics Statement

All necessary permits were obtained for the described regional soil inventory from the Qinghai Province Environmental Protection Bureau, which is responsible for the Three Rivers' Headwaters National Nature Reserve. No specific permissions are required by individuals since land in China belongs to the state. No endangered or protected species have been disturbed in our field sampling. The geographic information of sampling sites is provided in Table S1.

### Study Region

**Figure 1:**
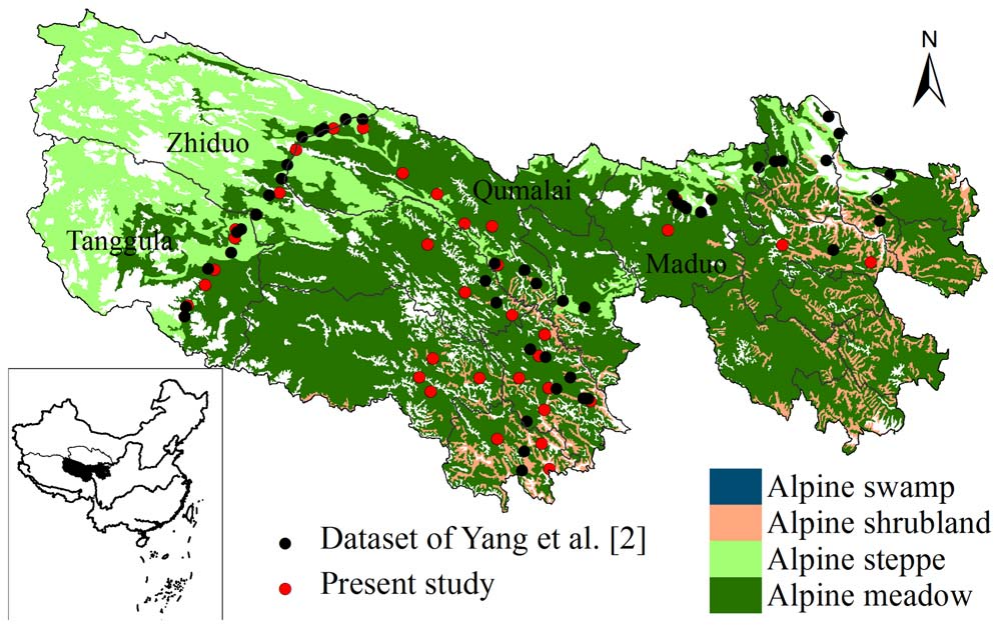
Soil sampling locations from our survey and the extracted dataset of Yang et al. [2].

The TRSR is composed of the water source region of the Yangtze River, Yellow River and Lancang (Mekong) River. The TRSR covers 30.23×10^4^ km^2^, which ranges in longitude from 89.75 to 102.38^o^E and in latitude from 31.65 to 36.20^o^N. Climate variation in the region is represented by a mean annual temperature range of −5.38 to 4.14°C, with mean annual precipitation ranging 262.2 to 772.8 mm, and annual evaporation from 730 to 1700 mm [16]. The elevation ranges from 3500 to 4800 m. Most of the region is dominated by alpine steppe and alpine meadow, with some areas of sparse alpine shrub and alpine marsh (Fig. 1). Alpine steppe is dominated by hardy perennial xeric herbs such as *Stipa purpure*, *Carex moorcrofii* and *Dalea racemosa*. Alpine meadow is dominated by *Kobresia pygmaea*, *K. humilis* and *K. tibetica*. Alpine shrub is dominated by *Salix oritrepha var. amnematchinensis* (L.). Alpine marsh has formed in permanently waterlogged areas or where the soil has been over-saturated, and supports hardy perennial hydro-philous or hydro-mesophytic herbs such as *K. tibetica* [17]–[18]. Soils in this region are shallow, with a depth of about 30–50 cm [19].

### Field Sampling and Laboratory Measurements

A total of 298 soil profiles from 35 sites were sampled from August to September 2006 and 2008 (Fig. 1). Of the 35 sites, 9 were alpine steppe, 21 were alpine meadow, 2 alpine shrubland and 3 alpine marsh (Table 1). At each site of alpine steppe and meadow, eight soil profiles along a 200 m transect were collected. For alpine shrub and marsh, 11 and 12 soil profiles were assigned to each transect, respectively. Soil samples were collected from each profile at every 10 cm to a depth of 30 cm. These soil samples were then mixed to yield one composite sample for each depth interval. All 105 composite samples (35 sites × 3 soil depths) were later air-dried, sieved (2 mm), and analyzed for SOC (measured with a Shimadzu 5000 SOC analyzer, Kyoto, Japan). Bulk density was taken using intact cores of 100 cm^3^ for each depth in the soil profile. The fine earth (< 2 mm) and stone content were weighed by oven-drying at 105°C for 48 h in the laboratory. The bulk density of the fine soil was calculated after correction for the mass of stone fragments. Then, bulk densities within each site were averaged for each depth. SOC densities (kg C m^−2^) were calculated in each depth interval as the product of soil C concentration (%), bulk density (g cm^−3^) and fixed soil depth (10 cm). SOC density for the 30 cm profile was calculated by summing stocks for the individual 10 cm layers.

**Table 1:** The study area, distribution of sampling sites and soil organic carbon density by grassland type in the Three River Source Region.

Grassland type	Area (10^4^km^2^)	Proportion (%)	No. of sample sites	Soil organic carbon density (kg C m^−2^)		
				0–10cm	10–20cm	20–30cm
Alpine meadow	20.18	67.9	21	2.93±0.83	2.44±0.61	2.02±0.53
Alpine steppe	7.52	25.3	9	1.71±0.51	1.57±0.38	1.48±0.35
Alpine shrubland	2.03	6.8	2	3.68±0.39	3.01±0.22	2.52±0.34
Alpine marsh	-	-	3	3.84±0.50	3.07±0.36	2.69±0.98
Total	29.73	100	35			

### Comparison Dataset Collection

In order to identify the effect of sample size on estimates of soil C stocks and their uncertainties on a regional scale, we began with a search of the dataset previously collected by Yang et al. [2]. In this dataset, soil samples were collected from 135 sites in July and August 2001–2004. SOC densities at different depths (30, 50, and 100 cm) were determined at each site. Climate data were also examined using spatial interpolation from the records of 43 meteorology stations across the Tibetan Plateau. Site-specific information on location, soil texture and grassland type are available in the dataset. From this dataset, we extracted records of 49 sites (23 in alpine meadow and 26 in alpine steppe) located in the TRSR for subsequent analysis.

### Data Analysis

One-way ANOVA was performed to compare the SOC density values of alpine grasslands in the current study with the corresponding dataset extracted from Yang et al. [2]. Geostatistical methods were used to examine the spatial variation in SOC across the study region. Experimental variograms were computed and the appropriate mathematical function was fitted to the semi-variogram. Maps of predicted SOC density were obtained for the study region using ordinary kriging integrated with the parameters of the appropriate variogram model. We then overlaid the 1:1000 000 vegetation map of the Tibetan Plateau (Chinese Academy of Science 2001) on the SOC density prediction maps to determine the SOC densities for each grassland type. Based on the statistics on SOC density, the regional SOC stock in a given depth interval can be calculated by the following equations:



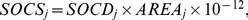
(1)




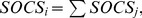
(2)


where SOCS_j_ is the SOC stock (Pg), SOCD_j_ is the SOC density (kg m^−2^), and AREA_j_ is the area (m^2^) for each grassland type *j*. SOCS_i_ is the regional SOC stock in layer *i* (0–10 cm, 0–20 cm and 0–30 cm). Based on the statistics on SOC density estimated using the formulas above, the average value and corresponding standard deviation were estimated using a Monte Carlo approach with 10 000 iterations. From these 10 000 runs, we obtained the average value and the 2.5 and 97.5 percentiles as the final estimate of the mean and of uncertainty (i.e., 95% confidence interval). A percentage uncertainty was estimated based on half of the 95% confidence interval divided by the average SOC stock estimate. Furthermore, we tested whether the number of sampling sites in the current study was sufficient to detect a 10% uncertainty of the SOC stock estimate with power analysis. All power calculations were based on a 0.05 probability of Type I error.

The data were analyzed using different software packages. The descriptive statistical parameters and statistical analyses were performed with SPSS 16.0 (SPSS Inc., USA). Spatial analyses were performed using ArcGIS 9.3 (ESRI Inc., USA). Monte Carlo simulation was calculated using RiskAMP software (http://www.thumbstacks.com/) added in Microsoft Excel 2003 (Microsoft Corporation, USA). Power analysis was conducted using the PROC MIXED procedure of SAS (SAS Institute, 2001).

## Results

### Overall Patterns of SOC Density

The experimental variogram for SOC revealed an evident spatial structure at the regional scale, with a nugget effect of about 15% (Table S2). This was reflected in the maps of the kriged estimates. The predicted value of SOC density decreased from the southeastern to the northwestern areas (Fig. S1). Spatial variation in SOC values was also observed for soil profiles. Table 1 illustrates the results, showing clearly that more C was stored in the top of the profile than at depth. Generally, alpine marsh soils had the largest SOC density at the three soil depths, followed by alpine shrubland and alpine meadow, and then alpine steppe.

### Regional SOC Stock and its Uncertainty

**Figure 2:**
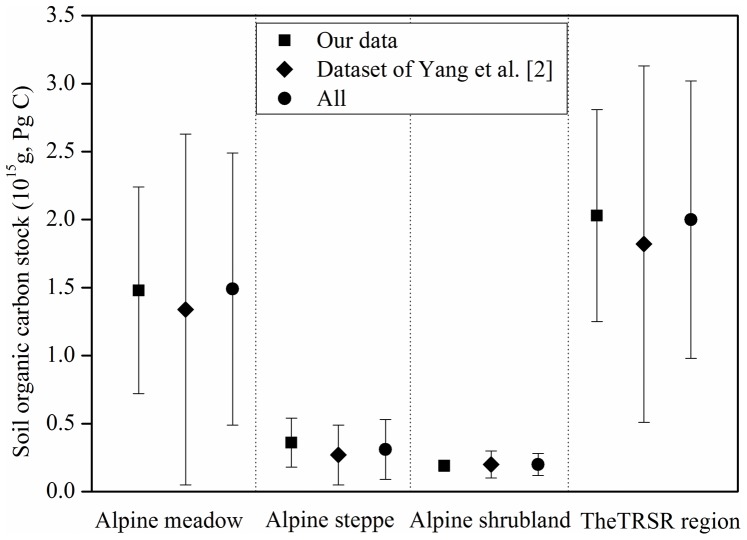
Mean SOC stocks with uncertainty estimates (95% confidence intervals) for alpine grasslands in the Three Rivers Source Region from our data, the dataset of Yang et al. [2], and the pooled dataset.

There were clear differences in total SOC stocks between grassland types (Fig. 2). Alpine meadow soils had the largest SOC stock, accounting for about 73% of the regional SOC stock for the three soil depths because of its extensive area (Table 1). By contrast, alpine marsh soils had the smallest areal extent, and lowest C stocks. The SOC stocks for alpine steppe and alpine shrubland soils were 0.36 and 0.19 Pg C, respectively. When the grassland area is examined overall, the estimate of the total stock for the TRSR is 2.03 Pg C. The 95% confidence interval around this estimate is 1.25 to 2.81 Pg C. Alpine meadow soils contributed most to the uncertainty in the regional C stock estimate (Fig. 2).

### Power Analysis of SOC

**Figure 3:**
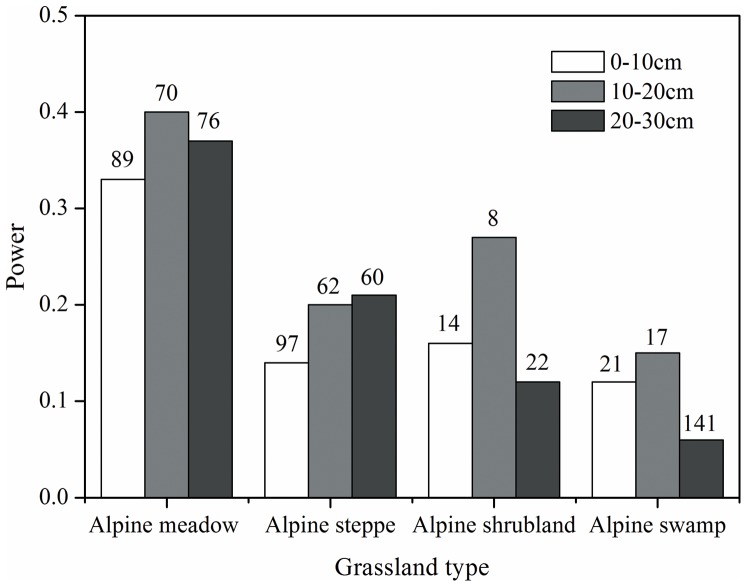
Probability (power) of detecting a 10% uncertainty in soil organic carbon density with a 0.05 level of significance for grassland types at three soil depths. The numbers above each bar represent the number of sampling sites needed to be taken at three depths for each alpine grassland to detect the tolerated uncertainty with 90% probability.

Following Allen et al. [6], we used tolerable uncertainty of 10% above and below the mean of SOC density. Fig. 3 shows the statistical power to detect a deviation within 10% uncertainty for our current study. The probabilities of detecting the tolerable uncertainty were extremely low for all grassland types at three soil depths. Alpine meadow had the largest sample size but had less than 40% chance of detecting the tolerable uncertainty. The statistical power was much lower for the other three grassland types. There were clear differences in the power values between soil depths. The larger power values were observed in the subsurface layers (10–20 and 20–30 cm) for alpine meadow and alpine steppe, while the other grassland types had the lowest values at 20–30 cm soil depth. Generally, the required number of sites to detect a deviation of 10% uncertainty with a statistical power of 0.90 was much higher for alpine meadow and alpine steppe than for the other grassland types. The power analyses showed that 90 sample sites are required to meet the tolerated uncertainty for estimating mean soil C stocks for alpine meadow, while 30 sites would be adequate for alpine shrubland at three soil depths. In comparison, about 100 sites would be necessary for alpine steppe at the surface 0–10 cm depth and 150 sites for alpine marsh at a deeper depth (20–30 cm).

## Discussion

### Comparisons of SOC Density with Earlier Observations

**Figure 4:**
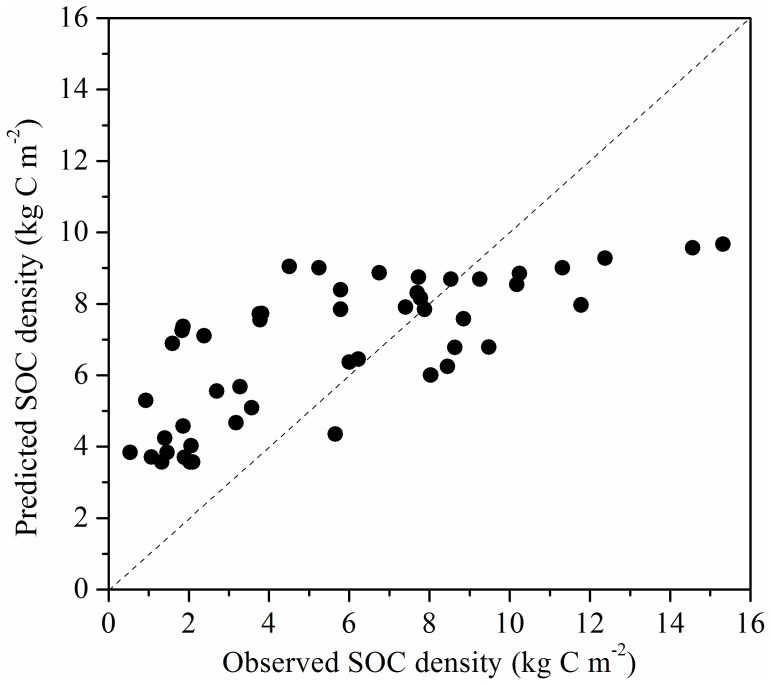
Predicted SOC densities by kriging of our data against observed values of Yang et al. [2].

**Figure 5:**
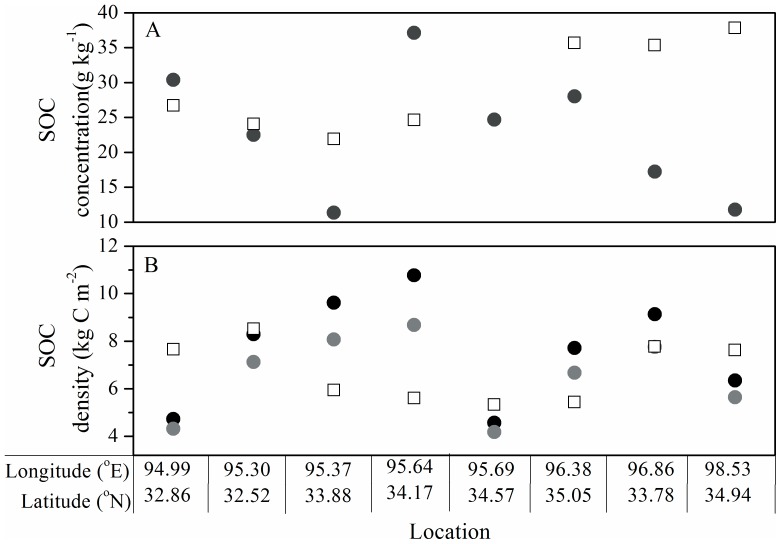
Comparisons of soil organic carbon concentration (A) and density (B) between present and previous studies. The present SOC values (open squares) were extracted from our kriged prediction map. The dark grey circles (A) indicated the measured SOC concentration values from previous study, while the previous corresponding SOC densities (B) were re-calculated using different bulk density values derived from pedotransfer functions of Yang et al. [22] (dark circles) and Zhong et al. [23] (gray circles), since bulk density was not measured in the previous study [21].

Our current estimates (Table 1) were comparable to that reported by Guo et al. [20] (alpine steppe = 3.88 kg C m^−2^; alpine meadow = 5.18 kg C m^−2^ for 0–30 cm). To compare regional datasets, data points geographically distributed in the TRSR were extracted from the dataset of Yang et al. [2]. SOC densities derived from our dataset and the extracted dataset were compared for alpine steppe and alpine meadow. We found that there were no significant differences (alpine steppe *t*  =  0.132, *P*  =  0.895; alpine meadow *t*  =  −1.175, *P*  =  0.246). We also compared the differences in SOC density estimates after kriging of our data vs. Yang et al. [2]. By contrast, local estimates were relatively divergent (Fig. 4). Likewise, SOC values (both concentration and recalculated density) were extracted from our prediction maps for site-specific comparison with previous studies [21]. There were large differences in SOC concentration and density at the small scale level (Fig. 5). These results indicated that regional estimates of SOC are very similar but local predictions based on kriging are not. Part of this variation of SOC density could be explained by varying methods used to determine soil bulk density. Because bulk density was ignored in previous studies, we estimated the bulk density using two pedotransfer functions for alpine grassland developed by Yang et al. [22] and Zhong et al. [23]. As shown in Fig. 5, there were relatively large differences in the SOC densities calculated using different bulk estimates. Some studies have shown that indirect bulk density estimates based on pedotransfer functions can lead to errors from 9% up to 36% of the SOC density [24], [25]. The most potential explanation for this deviation in SOC values may be attributed to the current sampling regime, which is not intensive enough to reveal the spatially explicit patterns of SOC in the study area. In spite of strongly spatial autocorrelation at the regional scale, the experimental variogram showed a relatively large nugget effect (15%), indicating that there was considerable short range variability.

### Sample Size Effect on Estimate Uncertainty

Despite a relatively large number of detailed surveys of grassland soil C stocks undertaken to date on the Tibetan Plateau, calculated errors remain relatively high, largely due to insufficient sample size and great sample variance [2]. Our analysis showed that the largest power value observed in any of the grassland types or soil depths exceeded 50%, and more than six times as many sites as those used in our survey were required to detect a 10% uncertainty in regional SOC stocks (Fig. 3). Hence we expected estimated uncertainty in SOC stock would decrease with increasing sample size. However, the estimated uncertainty of regional SOC stocks was not reduced when the two datasets were pooled together (Fig. 2). This result is perhaps unsurprising given that the spatial distribution of sampling sites across the study region was different between the two datasets. Because of bad weather and inaccessibility to vehicles, the previous survey by Yang et al. [2] was mainly conducted along the major roads, while more observations were distributed in the Lancang (Mekong) river basin in our field survey (Fig. 1). Areas that were intensively sampled captured local-scale spatial heterogeneity [26]. However, a sparse sampling distribution increases the standard deviation of the SOC density due to their large spatial coverage. Therefore, increasing sample size only without considering spatial representativeness would not necessarily reduce the level of uncertainty and improve statistical power.

### Implication for Sampling Design

Given the large uncertainty in estimation of regional SOC stocks, there is considerable interest in quantifying the magnitude and designing an efficient sample scheme to reduce uncertainty [26], [27]. One way of improving the efficiency and precision of a future survey is to tailor the sample size to the expected variability in soil C stock [28]. An additional ca. 200 sample sites across the TRSR would be required for accurate C stock estimate within 10% uncertainty, which would greatly increase costs and survey effort. Previous soil C research has produced a wealth of information that can be synthesized into a comprehensive and quantitative dataset, which offers an alternative that is likely to decrease sample size requirements for a subsequent inventory [7]. However, we found that almost all current studies concentrated sampling in easily accessed areas along the main roads on the Tibetan Plateau, while large areas that are poorly accessible due to limited road networks or poor security were substantially underrepresented [2], [3], [20], [23]. Future sampling effort on the Tibetan Plateau would be better directed to exploring these underrepresented areas, such as the northern Tanggula Mountains and the Hoh Xil region. However, a sound sampling design may be impractical and prohibitively expensive. Therefore, future inventories should incorporate the use of geoinformatics (GIS/Remote Sensing) tools or extrapolative spatial models of regional C stock estimates, as these have the potential to reduce costs [2], [29], [30]. As the greatest uncertainty in the regional SOC estimate originated from the variance in SOC values assigned to alpine meadow (Fig. 2), a number of additional sample sites to increase representation of alpine meadow soils would significantly reduce the uncertainties in regional soil C stock estimates in the TRSR.

## Conclusions

This work presents, to the best of our knowledge, the first regional estimate of SOC stock in the TRSR region. The SOC stored in alpine grassland soils of the TRSR region at 0–30 cm depth ranged from 1.25 to 2.81 Pg C at 95% confidence, with a mean of 2.03 Pg C. We observed that the largest source of uncertainty affecting regional SOC estimates derives from alpine meadow soils. SOC stocks varied with grassland type. Mean SOC stocks were 0.36 and 0.19 Pg C in alpine steppe and alpine shrubland, respectively. Approximately 73% (about 1.48 Pg C) of the regional SOC storage occurred in alpine meadow, which covers about 68% of the grassland area. Our result also indicated that uncertainty in the SOC stock estimates did not reduce when our dataset was pooled with the extracted dataset of Yang et al. [2], even though this provided more than twice as many sample sites as those in our survey. This most likely resulted from the underrepresentation of soils sampled in large areas that are relatively inaccessible in the northwest and southeastern part of the TRSR. Therefore, improvement in regional SOC stock estimates requires the addition of a number of sampling sites targeted to these known gaps and to address the high variability of estimated SOC stocks in alpine meadow soils.

## Supporting Information

Figure S1 **Spatial distribution of soil organic carbon density (SOCD) values for soils at (a) 0–10 cm, (b) 0–20 cm, and (c) 0–30 cm depth.**
(TIF)

Table S1 **Location of the sampling sites.**
(DOCX)

Table S2 **Parameter estimation of the fitted variogram of Gaussian models.**
(DOCX)
