# Supplementary material for: Alpine Grassland Soil Organic Carbon Stock and Its Uncertainty in the Three Rivers Source Region of the Tibetan Plateau
Source: PLoS One. 2014 May 12;9(5):e97140. doi: 10.1371/journal.pone.0097140 (PMC4018262; doi:10.1371/journal.pone.0097140)
Supplement: Table S1 — Location of the sampling sites. (DOCX) [file pone.0097140.s002.docx]

**Table S1**. Location of the sampling sites.

| **Site** | **Lat. ^o^N** | **Long. ^o^E** | **Site** | **Lat. ^o^N** | **Long. ^o^E** |
| --- | --- | --- | --- | --- | --- |
| 1 | 33.34 | 91.89 | 19 | 32.88 | 94.97 |
| 2 | 33.58 | 92.08 | 20 | 33.09 | 95.12 |
| 3 | 33.75 | 92.17 | 21 | 32.93 | 95.74 |
| 4 | 34.11 | 92.38 | 22 | 32.04 | 96.74 |
| 5 | 34.38 | 92.62 | 23 | 32.31 | 96.04 |
| 6 | 34.64 | 92.89 | 24 | 32.89 | 96.64 |
| 7 | 35.11 | 93.04 | 25 | 32.66 | 96.61 |
| 8 | 35.38 | 93.50 | 26 | 32.97 | 96.25 |
| 9 | 35.42 | 93.89 | 27 | 33.22 | 96.48 |
| 10 | 34.99 | 94.48 | 28 | 33.45 | 96.53 |
| 11 | 34.81 | 94.96 | 29 | 32.79 | 97.19 |
| 12 | 34.53 | 95.36 | 30 | 32.30 | 96.61 |
| 13 | 34.53 | 95.72 | 31 | 34.64 | 98.03 |
| 14 | 34.13 | 95.84 | 32 | 34.97 | 98.11 |
| 15 | 33.81 | 95.45 | 33 | 34.20 | 92.38 |
| 16 | 34.27 | 94.90 | 34 | 34.56 | 99.55 |
| 17 | 33.62 | 96.09 | 35 | 34.42 | 100.72 |
| 18 | 32.74 | 95.13 |  |  |  |
