# Supplementary material for: Alpine Grassland Soil Organic Carbon Stock and Its Uncertainty in the Three Rivers Source Region of the Tibetan Plateau
Source: PLoS One. 2014 May 12;9(5):e97140. doi: 10.1371/journal.pone.0097140 (PMC4018262; doi:10.1371/journal.pone.0097140)
Supplement: Table S2 — Parameter estimation of the fitted variogram of Gaussian models. (DOCX) [file pone.0097140.s003.docx]

**Table S2.** Parameter estimation of the fitted variogram of Gaussian models.

| Data | Nugget effect  (*C_o_*) | Sill  (*C*) | Nugget/sill  (*C_o_/C*) | Range  (*A_o_*, km) | *r^2^* |
| --- | --- | --- | --- | --- | --- |
| Our data | 4.30 | 28.59 | 0.15 | 1134 | 0.76 |
| Dataset of Yang et al. [2] | 0.99 | 17.56 | 0.06 | 226 | 0.81 |
| Pooled data | 3.94 | 15.18 | 0.26 | 395 | 0.83 |
